# Repetitive Transcranial Magnetic Stimulation Target Location Methods for Depression

**DOI:** 10.3389/fnins.2021.695423

**Published:** 2021-09-09

**Authors:** Min Zhang, Runhua Wang, Xin Luo, Si Zhang, Xiaomei Zhong, Yuping Ning, Bin Zhang

**Affiliations:** ^1^The Affiliated Brain Hospital of Guangzhou Medical University, Guangzhou, China; ^2^The First School of Clinical Medicine, Southern Medical University, Guangzhou, China; ^3^Guangdong Engineering Technology Research Center for Translational Medicine of Mental Disorders, Guangzhou, China

**Keywords:** MDD, brain stimulation, treatment, rTMS, location, DLPFC

## Abstract

Major depressive disorder (MDD) is a substantial global public health problem in need of novel and effective treatment strategies. Repetitive transcranial magnetic stimulation (rTMS) is a non-invasive and promising treatment for depression that has been approved by the U.S. Food and Drug Administration (FDA). However, the methodological weaknesses of existing work impairs the universal clinical use of rTMS. The variation of stimulated targets across the dorsolateral prefrontal cortex may account for most of the heterogeneity in the efficacy of rTMS. Many rTMS target location methods for MDD have been developed in recent decades. This review was conducted to assess this emerging field and to improve treatment outcomes in clinical practice.

## Introduction

Major depressive disorder (MDD) is a common and chronic mental disease that severely limits psychosocial function and impairs quality of life (Malhi and Mann, 2018). Further, more than one-third of MDD patients suffer from treatment-resistant depression (TRD) and fail to achieve remission of depressive symptoms after being treated with various strategies (Gaynes et al., 2008). Therefore, novel strategies to improve treatment outcomes for MDD patients need to be developed.

Repetitive transcranial magnetic stimulation (rTMS), which is approved by the U.S. Food and Drug Administration (FDA), is considered a safe and efficacious treatment for TRD (Levkovitz et al., 2015; Voigt et al., 2021). Although an increasing number of human studies indicate the efficacy of rTMS in MDD (O’Reardon et al., 2007; Fitzgerald et al., 2009a; Schutter, 2009), the response and remission rates of treatment are inconsistent (Loo and Mitchell, 2005). There are several possibilities: first, the heterogeneity of the patients such as age, disease duration time, degree of treatment resistance, and so on; second, different parameters such as the rTMS sequence, intensity, frequency, and so on; third, different location methods for the stimulated site. The standard rTMS treatment approach involves providing daily treatment sessions 5 days per week for a minimum of 4 weeks. The recommended intensity is 80–120% of resting motor threshold, and 4 weeks is a reasonable choice for the minimum duration of an adequate trial. As for the frequency, a recent study found no significant difference in groups between 5 and 10 Hz for treating MDD (Zhang et al., 2021). Since there are so many parameters combinations of the rTMS protocol, we are unable to cover all the possibilities. Instead, it is more practical to optimize the stimulated sites of coil placement in treatment. What is more, as the first step of rTMS treatment, the target location is so important that if the location is not accurate, the efficacy will be greatly compromised, no matter how well-designed the stimulated parameters are.

Repetitive TMS induces neuronal depolarization of a targeted brain region by applying an adjustable frequency of magnetic stimulation to generate a therapeutic effect (George et al., 2013). Prefrontal brain regions, in particular, the right and left dorsolateral prefrontal cortex (DLPFC), have been a focus of imaging studies in MDD (Kaiser et al., 2015). Neuroimaging studies have established the imbalance hypothesis of MDD, which postulated prefrontal asymmetry related to relative hypoactivity in the left DLPFC and relative hyperactivity in the right DLPFC (Mayberg et al., 1999; Rogers et al., 2004; Li et al., 2015). Therefore, high-frequency rTMS (HF-rTMS) and low-frequency rTMS (LF-rTMS) are often applied over the left and right DLPFC to facilitate or suppress brain activity respectively. Further, DLPFC is a key element of many high-order brain functions which relate to cortical and sub-cortical regions structurally and functionally (Xue et al., 2017). In summary, stimulating DLPFC may help to improve these brain functions and treat the associated brain disease such as depression. Though DLPFC is the most widely stimulated site for depression, and the efficacy has been confirmed by numerous previous studies (Fitzgerald, 2020), we should realize that other stimulated sites may also be developed. Downar and Daskalakis (2013) suggested that dorsomedial prefrontal cortex (DMPFC), ventromedial prefrontal cortex (VMPFC), orbitofrontal cortex (OFC), ventrolateral PFC (VLPFC), frontopolar cortex, and others also showed effective theoretical targets for rTMS in depression. However, the optimal type of stimulation parameters for these regions may be different, and with the DLPFC approved by FDA with a recommended parameters protocol, the current review mainly focuses on the studies with the stimulating site of DLPFC.

Though DLPFC is the dominant stimulated site for depression at present, it is a large brain region, and it is not clear which specific part is involved in MDD. Thus, remission rates of MDD may vary because efficacy may be associated with the specific stimulated site across the DLPFC (Siddiqi et al., 2020). Therefore, it is essential to accurately locate the specific targets across the DLPFC related to depression symptoms in order to improve the therapeutic effect of rTMS on MDD. Generally, there are two types of rTMS target location methods for depression: scalp-based measurement and neuroimaging-based targeting measurement. In the current review, we summarize the development of rTMS target location methods for depression. Besides the remission rate and response rate, the convenience of practice among studies of different location methods is also included to evaluate the current location methods. We try to make rough comparisons among them and provide some new insight into the improvement with rTMS in clinical practice.

## Scalp-Based Measurement

### 5-cm Method

In 1995, George et al. (1995) first proposed a “standard procedure” for positioning the coil to the DLPFC based on the Talairach atlas measurement. The hot spot on the primary motor cortex was searched by evoking a motor response of the contralateral abductor pollicis brevis muscle. Then the position of the DLPFC was measured along the scalp surface 5 cm forward. This procedure has been widely used since that time and is generally called the 5 cm method.

Many rTMS trials and MDD studies are performed using this method (Schutter, 2009). However, the 5 cm method does not take variation in skull shapes or brain anatomies among individuals into consideration, so it leads to stimulation of brain fields around the DLPFC, such as BA6 and BA8, in many patients (Herwig et al., 2001). Therefore, accuracy of the method is questioned by some researchers. Nauczyciel et al. (2011) found that the coil was not placed at the recommended target in 54% of depressed patients when using the 5 cm method for positioning. The average deviation between the position of the coil and the real target is 20.4 mm, detected by magnetic resonance imaging (MRI). Herwig et al. (2001) found that only 31.8% of the subjects were correctly targeted by the 5 cm method. Herbsman et al. (2009) reported that stimulating more lateral and anterior sites of the DLPFC acquired greater efficacy than using the traditional 5 cm method. Based on the similar results of the increasing studies (Brunoni et al., 2017), some researchers moved 5.5 or 6 cm forward from the hot spot to stimulate the critical DLPFC target, replacing the 5 cm method (Yesavage et al., 2018; Trapp et al., 2020).

### International 10–20 System

The international 10–20 system, a method for standardized placement of electroencephalogram (EEG) electrodes, has been increasingly applied for coil positioning in rTMS studies (Herwig et al., 2003b; Fitzgerald et al., 2009b; Rusjan et al., 2010). The 10–20 system is based on identifying anatomical landmarks such as nasion, inion, and preauricular points, with the consecutive placement of the electrodes at fixed distances from these points. Theoretically, compared to the 5 cm method, the 10–20 system considers variations of skull shapes and can make a rough correlation between external skull locations and underlying cortical areas. Herwig et al. (2003b) found that F3 of the 10–20 system can be used to target the DLPFC and has approximately 50% specificity. Other positions, such as points between AF3 and F3, are also detected (Fitzgerald et al., 2009b).

It will take time for beginners to use the 10–20 system due to the numerous measurements and calculations. Thus, Beam F3, which only uses three skull measurements to find the F3 position, has been developed (Beam et al., 2009). Compared to the traditional 10–20 system, 80% of patients’ F3 points can be precisely matched using the Beam F3 method. Furthermore, the average Beam F3 target is approximately 2.6 cm more anterolateral, which shows greater precision and reliability than the method of moving 5.5 or 6 cm forward from the hot spot (Trapp et al., 2020).

In general, scalp-based measurements, including the 5 cm method, 10–20 system, and Beam F3, were the most common target location methods in early TMS studies since they were easily applicable and cost less than neuroimaging methods. However, there is little evidence for the 5 cm method or 10–20 system electrode positions to certain anatomical locations (Rusjan et al., 2010).

## Neuroimaging-Based Targeting Measurement

### Structural MRI-Guided Method

An optically tracked frameless stereotaxic neuronavigation system based on structural MRI data has been developed. The neuronavigation system can locate the TMS coil to a specific anatomical site based on MRI data of the brain structure. More specifically, two ways can be applied by this method. Firstly, a standard head model in the neuronavigation system can be adopted without MRI scanning. However, the standard head model is possibly not fit for everyone, and it should only be an alternative when the patients have no other choice. The second way, which is also the more accurate way, is using the personalized structural MRI data of each participant to reconstruct the head model. The second way may match the 3D head model image to participants’ real head well. Compared with the traditional 5 cm method, structural MRI-guided neuronavigation localization is more accurate and consistent.

A prospective clinical trial found that rTMS with structural MRI-guided neuronavigation localization has significantly better clinical efficacy than rTMS guided by the 5 cm method (Fitzgerald et al., 2009b). However, a recent study found opposite results using prolonged intermittent theta-burst stimulation (piTBS) guided by the 5 cm method and the structural MRI-guided method as a monotherapy to investigate antidepressant efficacy. The study demonstrates that the structural MRI-guided coil positioning method may not be better than the 5 cm method (Li et al., 2020). In addition, a study showed that Beam F3 might provide a reasonable approximation to structural MRI-guided neuronavigation for locating the left DLPFC with discrepancies of less than 1.36 cm in 95% of subjects (Mir-Moghtadaei et al., 2015).

Thus, the existing studies do not entirely support the superiority of TMS targeting using structural MRI-guided methods, since the optimal stimulation site of the DLPFC remains unknown. The connection between the treatment area and other brain regions is ignored. Therefore, it is necessary to conduct more studies to investigate whether this method can significantly improve clinical efficacy in the future.

### PET-Based Method

Because structural MRI-guided methods have limitations, researchers have tried to develop new methods based on metabolism in the frontal cortex. Many functional neuroimaging studies have reported that regional cerebral blood flow (rCBF) decreases in the prefrontal, temporal, and anterior cingulate regions (ACCs) in patients (Kimbrell et al., 2002; Li et al., 2015). Furthermore, hypometabolism in the prefrontal region, related to depression, improves after successful treatment (Soares and Mann, 1997). This suggests that these hypometabolism areas that can be activated by rTMS may be potential targets for treatment.

A study that recruited 48 TRD patients compared the efficacy between positron emission tomography (PET)-guided rTMS and the 5 cm method (Paillere et al., 2010). They scanned patients with PET to find the most hypometabolic prefrontal area and determined the stimulation site in PET-guided rTMS. However, the results did not support the general hypothesis that HF-rTMS with prefrontal hypometabolism-related PET guidance increased antidepressant effects. Another PET study of 25 MDD patients found that the efficacy of stimulating the prefrontal hypometabolism site did not result in significantly different efficacy when compared to stimulating other sites independent of the metabolic state (Herwig et al., 2003a). Nonetheless, whether metabolism and anatomical characteristics of the DLPFC underneath the coil might account for an increase in rTMS efficacy needs further investigation.

### Functional MRI-Guided Method

#### Resting-State MRI-Guided Method

Neuroimaging studies of depression have significantly progressed in recent decades. Researchers have focused on investigating the role of abnormal functional brain networks in the pathophysiology of MDD (Connolly et al., 2013; Philip et al., 2018). Increasing studies of resting-state functional connectivity (FC) in MDD suggest that depression may be considered a disorder of the brain network (Kaiser et al., 2015). This meta-analysis indicated that depression associated with abnormal brain network models includes various cortical and subcortical brain regions. Some clinical studies have shown that rTMS can affect the stimulated site and a transsynaptic network of interconnected areas (Tik et al., 2017; Eshel et al., 2020). FC might be used to identify an optimal TMS target for use in MDD patients (Wang et al., 2019; Cash et al., 2020).

The resting-state MRI-guided method was developed based on the theories that alternations of the neural circuit commonly exist in psychiatric disorders and that alternations could be revised after successful treatment (Ge et al., 2017; Scibilia et al., 2018; Rudebeck et al., 2019). Fox et al. (2012) first reported that the efficacy of rTMS for MDD is associated with FC between the subgenual anterior cingulate cortex (sgACC) and DLPFC. Since then, an increasing number of studies have suggested that the most anticorrelated FC between a sub-region of DLPFC and sgACC was associated with better anti-depressant treatment (Fox et al., 2013; Blumberger et al., 2018; Jing et al., 2020; Fitzgerald, 2021). For instance, Hadas et al. (2019) suggested that hyperactivity in the subgenual cingulate cortex (SGC) was anticorrelated with activity in the DLPFC. Furthermore, they found that DLPFC-SGC connectivity was correlated with symptom improvement, indicating that the DLPFC-SGC connectivity might be a marker of rTMS treatment. Other stimulation sites, such as FC between the pregenual ACC and DLPFC, may also be a connectivity-based targeting strategy for focal brain stimulation that might be used to optimize clinical response (Zhou et al., 2017; Jing et al., 2020).

Blumberger et al. (2018) located the left DLPFC by reverse coregistration from the Montreal Neurological Institute (MNI) stereotaxic coordinate, which Fox suggested as the optimal target (Fox et al., 2012). Furthermore, they found that the response rate of efficacy was between 47 and 49%. Although they found promising clinical improvement, the target may not be suitable for every patient based on group-level connectivity that ignores individual differences in brain FC. Some researchers think that patients may obtain better efficacy if stimulation can be applied on sites based on their FC between the sgACC and DLPFC (Downar and Daskalakis, 2013; Fox et al., 2013; Singh et al., 2019). In one clinical trial with a small sample size, Cole et al. (2020) applied 10 intermittent burst stimulation (iTBS) sessions per day to the region of the left DLPFC, which was the most anticorrelated to the sgACC based on the FC of each patient. Personalized left DLPFC targets were generated using a series of complicated MRI analysis technologies for each participant, based on their baseline resting-state MRI data. 90.48% of the patients achieved remission of depression symptoms after only 5 days of treatment.

#### Task-State MRI-Guided Method

Although there are many location methods, we have no idea about the actual activity of the prefrontal cortex when we apply rTMS. However, we can find the activated area if we use task-state MRI to regulate DLPFC activity in real-time. Neacsiu et al. (2018) used task-state MRI to locate rTMS targets, and five patients with depression were given personalized colored phase stimulation. The left frontal neural network, which can induce peak activation by the task, was used as the specific rTMS target. The Hamilton Depression Rating Scale score significantly decreased with 4 weeks of rTMS treatment.

Another trial localizes the DLPFC using a “2 back” working memory task that can activate frontoparietal networks, including BA46 (Plichta et al., 2012). BA46, which can induce peak activation, is used as a stimulation target. However, there was no significant difference between the rTMS and sham groups. The task-state MRI-guided method does not obtain significant and consistent clinical efficacy, and it is a time-consuming and expensive method. It is necessary to conduct further studies to develop a better task that can activate specific areas of the DLPFC and increase efficacy.

## Discussion

Neurostimulation technologies such as rTMS have great potential as enduring, brain-based interventions for MDD. However, there are some unknowns regarding optimal stimulation sites and parameters for MDD patients receiving rTMS that impair the clinical efficacy of this technology (George et al., 2009; Wassermann and Zimmermann, 2012). Effectiveness studies of the location methods for stimulation sites have been performed to address these unknowns. In 1995, the 5 cm method was first proposed as a standard procedure to locate stimulation targets of the DLPFC quickly. However, this location method is not precise enough because it does not consider the difference in individuals’ skull shapes and anatomies. The 10–20 system overcomes some limitations of the 5 cm method, but it cannot ensure the relationship between the stimulated sites on the skull and the brain underneath. Although these two scalp-based methods are commonly used due to accessibility, the accuracy of positioning is questionable. The structural MRI-guided method enables researchers to locate the DLPFC through individualized anatomical images visually. However, it cannot ensure the optimal stimulation site of the DLPFC, which is associated with the efficacy of MDD. Therefore, PET-guided methods and resting-state and task-state MRI-guided methods have been developed to further identify specific sites in the DLPFC related to depressive symptoms through changes in metabolism, FC, or task activation. These neuroimaging-based targeting methods can ensure the location of the DLPFC and locate specific sites in the DLPFC that may play key roles in treating depressive symptoms.

The resting-state MRI-guided method is the most promising among all location methods, especially when it is based on individualized FC. Cole et al. (2020) use the stimulation protocol called Stanford Accelerated Intelligent Neuromodulation Therapy (SAINT) based on individualized FC, and the remission rate of MDD is up to 90.48%. However, we should be careful when promoting and applying SAINT since there is no control group. The placebo effect cannot be ignored with the high dose of pulses, intensive sessions, and 50-min intervals between sessions. In addition to determining the stimulated target by individualized FC, SAINT also applies a protocol with multiple novel features that may be difficult for other researchers and clinicians to repeat. Thus, it is uncertain to know how much the location and method contributed to the excellent outcomes. A former study using SAINT to treat only six patients reported that the response rate was approximately 80% (Williams et al., 2018). Whether the high response rate in these two studies results from individualized localization based on individualized FC or the combination of intensive stimulation sessions remains unclear and needs to be investigated in additional controlled studies. However, a recent review focusing on how neuroimaging was adopted to identify a better treatment target has supported that more effective TMS targets were functionally anti-correlated to the limbic system, such as the sgACC, which was the important role of resting-state MRI-guided method (Cash et al., 2020).

With an increasing number of studies, resting-state MRI-guided methods have also been applied to predict the efficacy based on the FC of patients at baseline. In addition to the FC between the DLPFC and sgACC (Weigand et al., 2018; Hadas et al., 2019), the effective baseline connectivity between the fronto-insular and salience networks (Iwabuchi et al., 2019), left DLPFC and striatum (Avissar et al., 2017), frontoparietal central executive network (CEN) and medial prefrontal-medial parietal default mode network (DMN) (Liston et al., 2014), and perigenual ACC and ventral medial prefrontal cortex (Long et al., 2020) have all been reported as predictive of rTMS efficacy. Efficacy prediction based on baseline FC can help clinicians identify patients who may have a better response to rTMS and save medical resources for patients who need more. However, it is necessary to overcome key technological problems, including improving functional MRI analysis techniques and decreasing the signal noise of MRI data, before resting-state MRI-guided methods are skillfully used.

Neuroimaging plays a key role in locating the position of DLPFC, but this method is relatively time-consuming, technology-dependent, and expensive. The 10–20 system takes the individual skull shape into account, which the 5 cm rule does not consider, and is cheaper and easier than neuroimaging. It might seem that an MRI scan was not that costly compared to the overall cost of treating MDD. However, some patients with poor economic conditions might still think it is too expensive. Furthermore, some clinics in developing countries do not have MRI machines or navigation systems. Even in hospitals with MRI machines, some patients cannot wait for the scan because there are so many patients who need it. Therefore, though neuroimaging-based location methods seemed to be better theoretically, we still supported that the international 10–20 system, especially the easier form Beam F3, may be a possible alternative when necessary.

In summary, there is still considerable scope to improve the efficacy of rTMS, including in the location methods of the stimulation site. There is no doubt that using brain imaging to improve the accuracy and specificity of TMS targets for depression is a trend. Although the 10–20 system is a practical alternative, we should realize that it cannot cover all the location methods when necessary. Though our review focuses on locating DLPFC in the rTMS treatment of depression accurately, it is important to note that the optimal practice of rTMS involves the precise location of DLPFC and other parameters such as the coil angle, stimulation intensity, frequency, and so on.

There are some limitations in the present review. Firstly, we did not quantitatively compare the effect sizes between different target location methods since few studies directly compared the efficacy of different target location methods. Second, this review did not compare the stimulated parameters simultaneously, which might also be essential factors for the efficacy of rTMS.

Although there have been considerable developments in rTMS areas in recent years, we still lack substantive prospective clinical trials demonstrating that we can improve overall clinical outcomes. This should be our focus for new research in this field now. A large sample number, randomized and controlled clinical trials with homogeneous MDD patients should be conducted to prospectively test and optimize resting-state MRI-guided location methods to evaluate their effectiveness compared with conventional scalp-based location methods. Importantly, research in locating the TMS method should be combined with studies in stimulated strategies of TMS such as intensity, frequency, durations, and so on, which together may promote the clinical outcome better.

## Author Contributions

All authors have contributed to the conception, editing, and revision of the manuscript, approved the final version to be published, and agreed to be accountable for all aspects of the work.

## Conflict of Interest

The authors declare that the research was conducted in the absence of any commercial or financial relationships that could be construed as a potential conflict of interest.

## Publisher’s Note

All claims expressed in this article are solely those of the authors and do not necessarily represent those of their affiliated organizations, or those of the publisher, the editors and the reviewers. Any product that may be evaluated in this article, or claim that may be made by its manufacturer, is not guaranteed or endorsed by the publisher.
